# Identification of Antibacterial Metabolites from Endophytic Fungus *Aspergillus fumigatus,* Isolated from *Albizia lucidior* Leaves (Fabaceae), Utilizing Metabolomic and Molecular Docking Techniques

**DOI:** 10.3390/molecules27031117

**Published:** 2022-02-08

**Authors:** Mai E. Hussein, Osama G. Mohamed, Ahlam M. El-Fishawy, Hesham I. El-Askary, Amira S. El-Senousy, Ahmed A. El-Beih, Eman S. Nossier, Ahmed M. Naglah, Abdulrahman A. Almehizia, Ashootosh Tripathi, Ahmed A. Hamed

**Affiliations:** 1 Pharmacognosy Department, Faculty of Pharmacy, Cairo University, Kasr el Aini St., Cairo 11562, Egypt; osama.mohamed@pharma.cu.edu.eg (O.G.M.); ahlam.elfishawy@pharma.cu.edu.eg (A.M.E.-F.); hesham.elaskary@pharma.cu.edu.eg (H.I.E.-A.); amira.elsenousy@pharma.cu.edu.eg (A.S.E.-S.); 2 Natural Products Discovery Core, Life Sciences Institute, University of Michigan, Ann Arbor, MI 48109, USA; ashtri@umich.edu; 3 Department of Chemistry of Natural and Microbial Products, National Research Centre, Dokki, Giza 12622, Egypt; aa.el-beih@nrc.sci.eg; 4 Department of Pharmaceutical Medicinal Chemistry and Drug Design, Faculty of Pharmacy (Girls), Al-Azhar University, Cairo 11754, Egypt; dr.emannossier@gmail.com; 5 Department of Pharmaceutical Chemistry, College of Pharmacy, King Saud University, Riyadh 11451, Saudi Arabia; anaglah@ksu.edu.sa (A.M.N.); mehizia@ksu.edu.sa (A.A.A.); 6 Department of Medicinal Chemistry, College of Pharmacy, University of Michigan, Ann Arbor, MI 48109, USA; 7 Microbial Chemistry Department, National Research Centre, 33 El-Buhouth Street, Dokki, Giza 12622, Egypt; ahmedshalbio@gmail.com

**Keywords:** *Albizia lucidior*, *Aspergillus fumigatus*, UHPLC–QTOF, antibacterial, DNA gyrase, topoisomerase IV

## Abstract

The rapid spread of bacterial infection caused by *Staphylococcus aureus* has become a problem to public health despite the presence of past trials devoted to controlling the infection. Thus, the current study aimed to explore the chemical composition of the extract of endophytic fungus *Aspergillus fumigatus*, isolated from *Albizia lucidior* leaves, and investigate the antimicrobial activity of isolated metabolites and their probable mode of actions. The chemical investigation of the fungal extract via UPLC/MS/MS led to the identification of at least forty-two metabolites, as well as the isolation and complete characterization of eight reported metabolites. The antibacterial activities of isolated metabolites were assessed against *S. aureus* using agar disc diffusion and microplate dilution methods. Compounds ergosterol, helvolic acid and monomethyl sulochrin-4-sulphate showed minimal inhibitory concentration (MIC) values of 15.63, 1.95 and 3.90 µg/mL, respectively, compared to ciprofloxacin. We also report the inhibitory activity of the fungal extract on DNA gyrase and topoisomerase IV, which led us to perform molecular docking using the three most active compounds isolated from the extract against both enzymes. These active compounds had the required structural features for *S. aureus* DNA gyrase and topoisomerase IV inhibition, evidenced via molecular docking.

## 1. Introduction

The bioprospecting of new antimicrobial products is a growing concern worldwide. Treatment of microbial infection caused by the ESKAPE group of pathogenic microbes, *Enterococcus*, *Staphylococcus*, *Klebsiella*, *Acinetobacter*, *Pseudomonas* and *Enterobacteriaceae*, is becoming difficult due to antimicrobial resistance (AMR) against existing antibacterial drugs [1]. Therefore, an exhaustive search for novel antimicrobials is of great importance to human health [2,3].

Endophytic fungi are organisms that colonize plant tissues. Some of them act in a symbiotic manner as they protect their host against pathogens through their secondary metabolism and, in turn, utilize the primary metabolites of plants for their maintenance and growth functions [4]. Endophytic fungi are a prolific producer of several FDA-approved drugs, like penicillin, vincristine, vinblastine, etc. [5,6,7].

The genus *Albizia,* of the family Fabaceae, consists of at least 150 species distributed over Africa, America and Asia [8]. *Albizia* species are traditionally used in Africa to treat rheumatism, cough, diarrhea and injuries [9]. Biological activities of *Albizia* species have been reported to possess antioxidant, anticancer, antidiabetic, anti-inflammatory, antibacterial and hepatoprotective properties [10]. *Albizia lucidior* (*A. lucidior*), commonly known as the Potka siris, is an Asian tree [8]. Chungtia villagers traditionally used it for the treatment of skin-related ailments [11]. The antibacterial activity of the ethanolic extract of *A. lucidior* roots was previously evaluated, showing moderate activity against both susceptible and MRSA *S. aureus* [11]. However, the chemical potential of endophytic fungi living symbiotically with this plant has not been explored yet.

Therefore, the isolation of endophytic fungi from the rarely studied plant, *A. lucidior*, allowed us to find novel fungal strains that may have a high chemical diversity with drug-like properties. In this study, we aimed to isolate secondary metabolites from the endophytic fungus *Aspergillus fumigatus* (*A. fumigatus*), isolated from leaves of *A. lucidior*. *A. fumigatus* fungus is able to produce several secondary metabolites belonging to different classes as polyketides, phenolics, triterpenes, sterols, sesquiterpenes, alkaloids, fatty acids, etc. [12], Based on previous reports, polyketides and metabolites containing the tetracyclic ring system exhibited diverse biological activities, such as cytotoxic [13,14] and antimicrobial [15,16] activities, that encouraged us to comprehensively evaluate the antimicrobial activities of isolated metabolites and investigate their mechanisms of action.

## 2. Results

### 2.1. Endophytic Fungi Isolation and Cultivation

The fungal strain reported here was one of fifty-four endophytic fungi isolated from the leaves of *A. lucidior*. Each isolated fungal strain was tested for its potential to exert antimicrobial activity using an agar disc-diffusion method (data are not shown). The most bioactive fungal species was further processed for identification. 

### 2.2. Identification of the Fungus Based on Phenotypic and Genotypic Characteristics

The colony morphology of endophytic fungus reached 1–2 cm diameter in 5 days and showed white with yellow reverse. Microscopically, the conidial heads were short columnar, the conidiophores were 3 μm in diameter, the vesicle was small with 7 μm in diameter and the conidia was broadly ellipsoidal (4 × 3 µm in size), coming from sterigmata (5 × 2 μm in size) (Figure S1). Additionally, the genomic DNA of the fungus was sequenced and the analysis of the ITS sequence was carried out using the BLAST tool to identify the similarity score and to calculate the statistical significance of the matches; the result established a very close similarity to that of *A. fumigatus*, with a homology of 99.50% (Figure S2). The phylogenetic analysis and the tree were composed using the neighbor-joining method to measure the evolutionary relationships of the obtained sequence and other similar sequences in the genebank database (Figure S3). Based on colony morphology, microscopic characteristics, the analysis of ITS rDNA sequence and phylogenetic characteristics, the isolate was identified as *Aspergillus fumigatus* and deposited in GenBank with accession no. MN519723.1.

### 2.3. UHPLC–QTOF Analysis of A. fumigatus Ethyl Acetate Extract

The EtOAc extract was analyzed in both positive and negative ion electrospray ionization (ESI) MS modes that led to the identification of forty-two metabolites belonging to different phytochemical classes. Metabolites were tentatively identified by comparing the accurate mass and fragmentation pattern with metabolites previously reported in the literature, as well as bioinformatics analysis using Sirius [17] and GNPS [18]. A representative chromatogram is presented in Figure S4. The retention times, identities, observed molecular weight and fragment ions for individual metabolites are shown in Table 1.

### 2.4. Isolation and Characterization of the Metabolites

**Figure 1:**
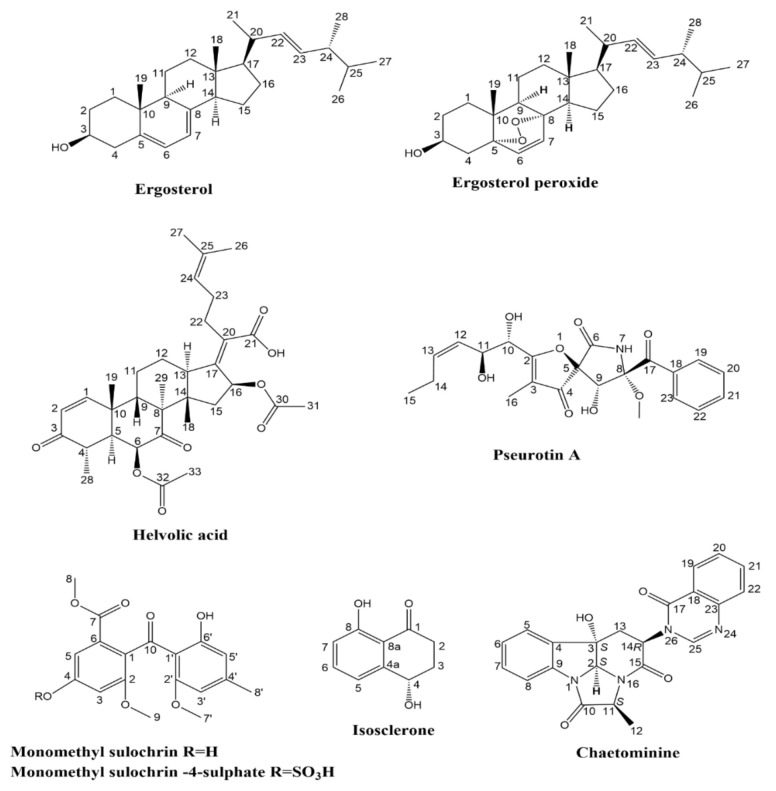
Structures of metabolites isolated from *A. fumigatus* ethyl acetate extract.

Chromatographic separation of *A. fumigatus* EtOAc extract yielded eight metabolites. The isolated metabolites were identified based on detailed 1D, 2D NMR and ESI-MS data compared with those reported in the literature (see supplementary file). Our studies resulted in identification of ergosterol [47], (22E)-5α,8α-Epidioxyergosta-6,22-dien-3β-ol (ergosterol peroxide) [48], helvolic acid [49,50], pseurotin A [51,52], monomethyl sulochrin [53], (4*S*)-isosclerone [54,55], monomethyl sulochrin-4-sulphate [33] and chaetominine [30] from the fungal strain (Figure 1). All these isolated metabolites have previously been reported as being isolated from *A. fumigatus* [33,56,57,58].

**Figure 2:**
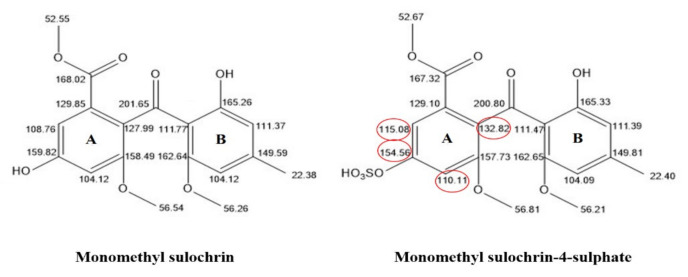
Difference in ^13^C-NMR chemical shifts data between monomethyl sulochrin and its sulphated derivative.

Monomethyl sulochrin-4-sulphate was obtained as a pale red amorphous powder with a brown fluorescence property under UV light at λ 254 and 365 nm. It turns red when derivatized with *p*-anisaldehyde/H_2_SO_4_ and heated at 110 °C. The ESI-MS spectrum showed a peak at *m*/*z* 425 [M − H]^−^ (Figure S5). The ^13^C-NMR data (Figure S6) and ^1^H-NMR spectrum (Figure S7) indicated the presence of four aromatic methine carbons (*δ*c 115.1/ *δ*_H_ 7.51, *δ*c 111.4/ *δ*_H_ 6.39, *δ*c 110.1/ *δ*_H_ 7.25, *δ*c 104.1/ *δ*_H_ 6.21 ppm); four oxygenated aromatic carbons (*δ*c 165.3, 162.7, 157.7, 154.6 ppm) and four aromatic carbons (*δ*c 149.8, 132.8, 129.1, 111.5 ppm). In addition, three methoxy carbons (*δ*c 56.8/ *δ*_H_ 3.74, *δ*c 56.2/ *δ*_H_ 3.36, *δ*c 52.7/ *δ*_H_ 3.70 ppm) and one methyl carbon (*δ*c 22.4/ *δ*_H_ 2.30 ppm) were observed. There was a carbonyl group and an ester group at *δ*c 200.8 and *δ*c 167.3 ppm, respectively. These spectral data were very similar to compound monomethyl sulochrin with a distinct deviation in ring A (Figure 2). A downfield shift was detected for H-3 and H-5 that appeared as doublets at *δ*_H_ 7.25 ppm (*d*, *J* = 2) and *δ*_H_ 7.51 ppm (*d*, *J* = 2), respectively, versus *δ*_H_ 6.68 ppm (*d*, *J =* 2 Hz) and *δ*_H_ 6.96 ppm (*d*, *J =* 2 Hz) in monomethyl sulochrin.

Also, two methyl protons of 8-OMe and 9-OMe were shifted downfield to *δ*_H_ 3.70 ppm (*s*) and *δ*_H_ 3.74 ppm (*s*), respectively. Furthermore, the ^13^C-NMR spectrum displayed downfield shifts at [*δ*_C_ 132.8 (C-1), 110.1 (C-3) and 115.1 ppm (C-5)] by 4.8, 6.0 and 6.3 ppm, respectively, whilst C-4 experienced an upfield shift of 5.2 ppm. The effect of *O*-sulfate as an electron-withdrawing group resulted in a decreased electron density (downfield shift) of the ortho and para carbons and an increased electron density (upfield shift) of the carbon carrying the sulphate group [59]. These shifts suggested that the sulfate group was most likely located at C-4 (Figure 2). This assessment was supported by HSQC (Figure S8) and HMBC (Figure S9). The HMBC spectrum revealed the following correlations: H-5 (*δ*_H_ 7.51) / *δ*_C_ 110.1 (C-3), 132.8 (C-1), 154.6 (C-4) and 167.3 (C-7); H-3 (*δ*_H_ 7.25)/ *δ*_C_ 115.1 (C-5), 132.8 (C-1), 154.6 (C-4) and 157.7 (C-2). The difference between observed ESI-MS data of monomethyl sulochrin (345 *m*/*z*) and monomethyl sulochrin-4-sulphate (425 *m*/*z*) in negative mode was 80 *m*/*z*, suggesting the presence of a sulfate moiety. Therefore, this compound was assigned as monomethyl sulochrin-4-sulphate (C_18_H_18_O_10_S). 

### 2.5. Biological Activity

#### 2.5.1. Antimicrobial Activity

The antimicrobial activities of the fungal extract and the isolated metabolites were screened using agar disc diffusion and microplate dilution methods. The obtained results (Table 2) revealed promising antimicrobial activity of the fungal extract against tested microorganisms. The data displayed the highest inhibition zones against *S. aureus* and *P. vulgaris*, with inhibition zones of 23.07 and 14.37 mm, respectively, compared to ciprofloxacin, with inhibition zones of 36.90 and 34.50 mm, respectively. In addition, the extract also demonstrated robust antifungal activity against *C. albicans*, with an inhibition zone of 17.13 mm, compared to nystatin, with an inhibition zone of 30.27 mm. Further antimicrobial evaluation of extract against *S. aureus*, *P. vulgaris* and *C. albicans* revealed the strong MIC values of 7.81, 15.63 and 15.63 µg/mL, respectively, compared to ciprofloxacin (MIC values of 0.63 and 1.25 against *S. aureus* and *P. vulgaris*, respectively) and nystatin (MIC value of 2.5 µg/mL against *C. albicans*). On the other hand, it showed moderate effects against *B. subtilis* and *E. coli* with MIC values of 31.25 and 62.50 µg/mL, respectively, and weak activity against *P. aeruginosa* and *A. niger* (MIC value of 125 µg/mL).

Based on the promising antibacterial activities of fungal extract, we proceeded with the characterization of bioactivity against *S. aureus* associated with isolated metabolites (Table 3). We observed that helvolic acid and monomethyl sulochrin-4-sulphate showed the highest inhibition zones of 33.00 and 26.56 mm, respectively, and the lowest MIC values (1.95 and 3.91 µg/mL, respectively). The antibacterial activity of these metabolites was higher than the fungal extract (MIC = 7.81 µg/mL) as expected, due to the lack of constitutive effect often displayed by complex extracts compared to pure entities. Ergosterol also exhibited considerable antibacterial activity with an inhibition zone of 14.10 mm and a MIC value of 15.63 µg/mL. 

#### 2.5.2. In Vitro Enzyme Assessment

The fungal EtOAc extract showed promising inhibitory activity towards DNA gyrase (IC_50_ = 0.86 µg/mL), compared to that of ciprofloxacin (IC_50_ = 0.51 µg/mL). Moreover, it revealed almost a two-fold increase in the suppression effect towards topoisomerase IV (IC_50_ = 1.23 µg/mL), compared to ciprofloxacin (IC_50_ = 2.15 µg/mL) (Table 4).

### 2.6. Molecular Modeling Study

**Figure 3:**
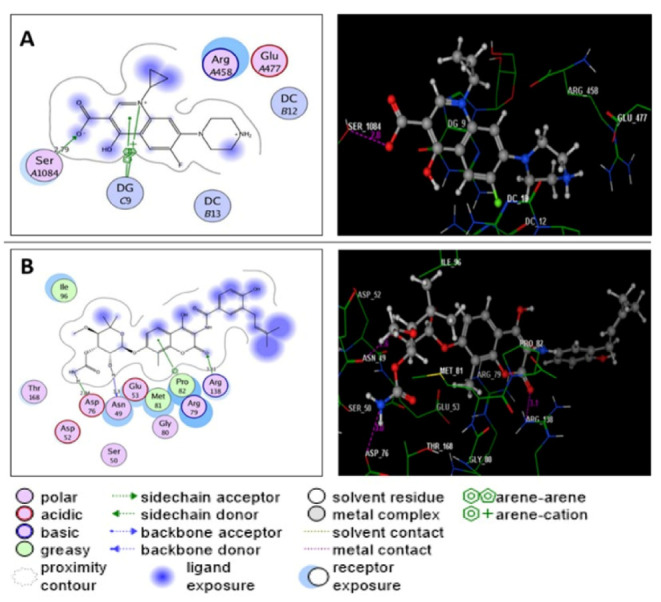
(**A**,**B**) diagram illustrate 2D and 3D binding patterns of the co-crystallized ligands ciprofloxacin and novobiocin within the ATP-active pocket of *S. aureus* DNA gyrase (PDB code: 2XCT) and topoisomerase IV (PDB code: 4URN), respectively. (Hydrogen bonds are illustrated as dashed lines; C atoms are colored gray, N blue and O red.)

**Figure 4:**
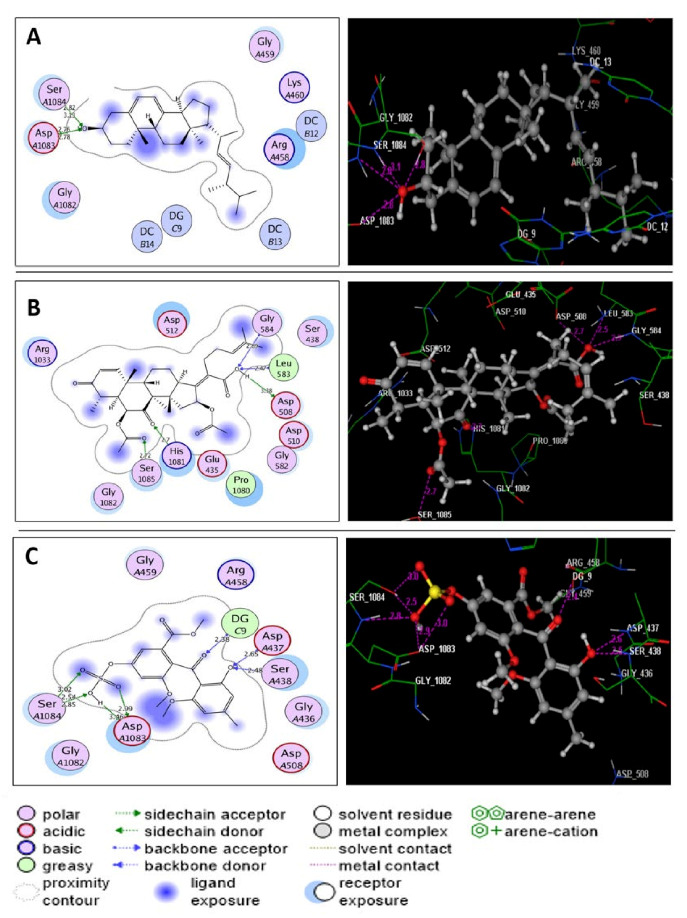
(**A**–**C**) diagrams illustrating 2D and 3D binding patterns of ergosterol, helvolic acid and monomethyl sulochrin-4-sulphate within the ATP-active pocket of *S. aureus* DNA gyrase (PDB code: 2XCT), respectively. (Hydrogen bonds are illustrated as dashed lines; C atoms are colored gray, N blue and O red.)

**Figure 5:**
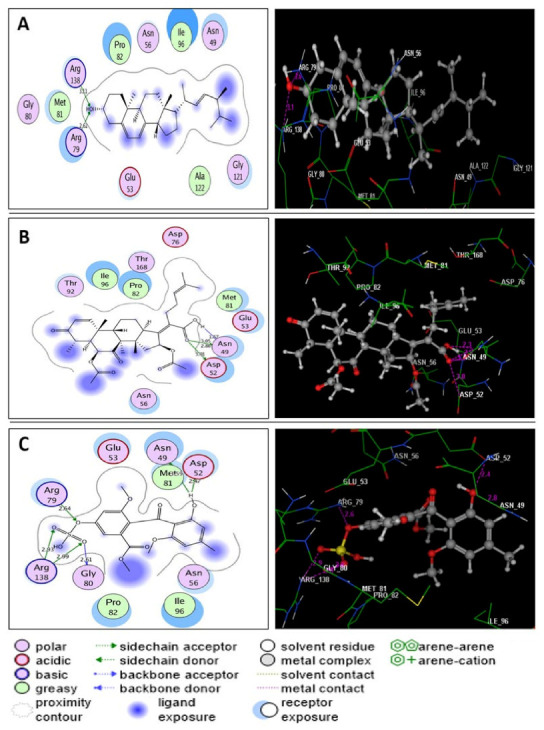
(**A**–**C**) diagrams illustrating 2D and 3D binding patterns of ergosterol, helvolic acid and monomethyl sulochrin-4-sulphate within the ATP-active pocket of *S. aureus* topoisomerase IV (PDB code: 4URN), respectively. (Hydrogen bonds are illustrated as dashed lines; C atoms are colored gray, N blue and O red.)

A docking simulation study of the three most active metabolites was performed to explain the observed promising potencies of the fungal extract against *S. aureus* DNA gyrase and topoisomerase IV and to explore the possible binding modes and interactions of these ligands within the active sites of both enzymes. First, the energy minimized ligands, ciprofloxacin and novobiocin, were re-docked in the active site of *S. aureus* DNA gyrase and topoisomerase IV (Figure 3A,B). Ciprofloxacin and novobiocin revealed energy scores of −7.22 and −7.60 kcal/mol at root mean square deviation (RMDS) values equal to 0.90 and 1.26, respectively. Furthermore, the isolated active compounds, ergosterol, helvolic acid and monomethyl sulochrin-4-sulphate, were docked into the ATP-active sites of *S. aureus* DNA gyrase (Figure 4A–C) and topoisomerase IV (Figure 5A–C), and the obtained docking data are recorded in Table 5 and Table 6.

Through detailed analysis of the acquired docking data, it was clear that ergosterol, helvolic acid, and monomethyl sulochrin-4-sulphate were fitted within the ATP-active site of *S. aureus* DNA gyrase with energy scores ranging from −7.40 to −8.83 kcal/mol (Table 5). Ergosterol displayed four H-bond acceptors between the hydroxyl oxygen and the sidechains of Asp1083 and Ser1084 (distance: 2.76, 2.78, 2.82 and 3.13 A°, respectively) (Figure 4A). Helvolic acid exhibited three hydrogen bonds between the hydroxyl group and the amino acids Asp508, Leu583 and Gly584 (distance: 3.38, 2.47 and 2.89 A°, respectively). Another two hydrogen bond acceptors appeared between oxygens of the carbonyl group at p-7 and acetoxy group at p-6, with the sidechains of His1081 and Ser1085 (distance: 2.70 and 2.72 A°, respectively) (Figure 4B). On the other hand, monomethylsulochrin-4-sulphate formed three H-bond acceptors between the oxygens of hydroxyl and carbonyl groups linked to the benzoyl moiety with Asp437, Ser438 and DG9 (distance: 2.65, 2.48 and 2.38 A°, respectively). Also, the sulfate group shared binding by forming five H-bonds with the sidechains of Asp1083 and Ser1084 (distance: 2.99, 3.46, 2.54, 2.85 and 3.02 A°, respectively) (Figure 4C).

Regarding docking within the ATP-active pocket of *S. aureus* topoisomerase IV (Figure 5A–C), all screened targets identified compounds from the fungal strain achieved promising binding with an energy score in the range −7.92 to −8.43 kcal/mol (Table 6). The hydroxyl oxygen of ergosterol exhibited H-bond acceptors with the sidechains of Arg79 and Arg138 (distance: 2.62 and 3.11 A°, respectively) (Figure 5A). The carboxylic group of helvolic acid established four H-bond donors with Asn49 and Asp52 (distance: 1.57, 2.86, 3.05 and 3.01 A°, respectively) (Figure 5B). While monomethyl sulochrin-4-sulphate formed six H-bonds, two of them were demonstrated between the hydroxyl proton and the sidechains of Asn49 and Asp52 (distance: 3.59 and 2.47 A°, respectively). The other four H-bonds were formed between the oxygen atom of the sulfate group and Arg79, Gly80 and Arg138 (distance: 2.64, 2.61, 2.93 and 2.99 A°, respectively) (Figure 5C).

**Figure 6:**
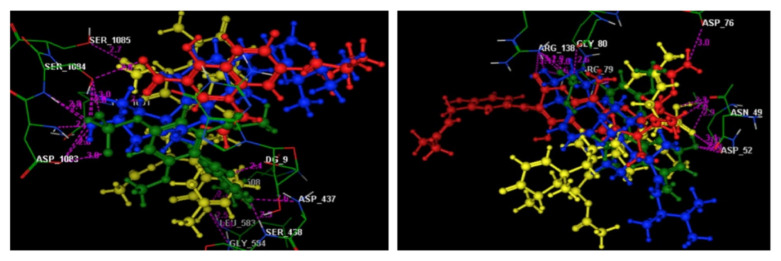
3D images of the superimposition between the docked ciprofloxacin or novobiocin (**red**) with helvolic acid (**yellow**), monomethyl sulochrin-4-sulphate (**green**) and ergosterol (**blue**) within the active sites of *S. aureus* DNA gyrase and topoisomerase IV (PDB codes: 2XCT and 4URN). (Hydrogen bonds are illustrated as dashed lines; C atoms are colored gray, N blue and O red.)

Finally, in a preliminary attempt to identify the mechanism of action of the promising inhibitory activities against *S. aureus* DNA gyrase and topoisomerase IV, the highly adapted overlap located in Figure 6 of the compounds screened within the binding sites of the two target enzymes, together with their original ligands ciprofloxacin and novobiocin, respectively, indicate that the tested compounds could behave similarly to the reference two.

## 3. Discussion

There has been growing interest in endophytic organisms isolated from plants, producing diverse secondary metabolites with different biological activities reported in recent literature [5,60]. Genus *Albizia* has been reported as a rich source of endophytic fungi [61] that have valuable bioactivities, including antimicrobial [62], cytotoxic [63] and antioxidant [64].

The isolation of endophytic fungi from *A. lucidior* has not been previously reported, so we bioprospected the isolation of the endophytic fungus from this plant with promising antimicrobial activity. Among fifty-four isolated endophytes, *A. fumigatus* extract showed the most promising broad-spectrum antimicrobial activity against *S. aureus*, *P. vulagris* and *C. albicans*. Furthermore, we also isolated and characterized at least eight metabolites previously reported from *A. fumigatus* (Figure 1) and carried out mass-spectrometry-based identification of forty-two metabolites (Table 1).

We also recorded antibacterial activities of isolated metabolites (ergosterol, helvolic acid and monomethyl sulochrin-4-sulphate) in addition to the fungal extract when tested against *S. aureus*. The antibacterial activities of ergosterol and helvolic acid and their potential mechanisms of action against *S. aureus* had previously been studied. However, the antibacterial activity of monomethyl sulochrin-4- sulphate and its probable mode of action was explored for the first time in this study.

Further, ergosterol displayed considerable antibacterial activity against *S. aureus* [65,66,67]. This activity could be associated with the hydroxyl group, which is essential for antibacterial activity based on its binding to the DNA-gyrase complex via hydrogen bonds as observed in the molecular docking study of thiazole derivatives [68]. 

Helvolic acid, which is a fusidane-type antibiotic, also showed potent inhibitory activity against *S. aureus* [33,39,69,70]. The carbon skeleton of helvolic acid has a similar skeleton to fusidic acid, with a different substitution pattern [71]. Fusidane-type antibiotics inhibit the translocation step of the protein synthesis [71,72]. According to structure–activity relationship studies, it was found that 17,20-double bond, α, β-unsaturated carboxylic acid and 16β-acetoxyl group are essential for observed antimicrobial activity [71,72]. Moreover, a recent study illustrated the bioactivities of helvolic acid derivatives on *S. aureus*, revealing that 3-keto and free C-6 hydroxyl groups are also important [69].

Sulfur-containing compounds are found in a wide variety of organisms [73,74,75]. For example, sulfated sterols from marine organisms showed antimicrobial activity against *S. aureus*, whereas the respective sterols were not active [75]. On the other hand, a new derivative of sulochrin, sulochrin dimers linked via a thioether bridge, exhibited significant anti-MRSA activity [73]. These findings suggested that the presence of sulfur is necessary for the observed antibacterial activity and supported our results for the antibacterial activity of monomethyl sulochrin-4-sulphate.

*S. aureus* is considered a major human pathogen that often causes skin infections, pneumonia, heart valve infections and device-related infections [76]. This prompted us to explore the possible mechanism of action of the fungal extract and its isolated metabolites against *S. aureus*.

DNA Gyrase and topoisomerase IV play an essential role in the regulation of bacterial cell cycle progression. DNA gyrase introduces negative supercoils into relaxed DNA, which is essential for DNA replication, elongation and transcription. In contrast, topoisomerase IV plays a crucial role in decatenating multiple linked chromosomes during DNA replication [77,78]. Based on the important functions of these enzymes, it makes them attractive targets for exploring more effective antimicrobials. Our results revealed that the fungal extract showed the potential to interact with both DNA gyrase and topoisomerase IV, which was observed to manifest through its inhibitory activity against *S. aureus*.

Prompted by the antibacterial results of isolated metabolites, ergosterol, helvolic acid and monomethyl sulochrin-4-sulphate were chosen for molecular docking against *S. aureus* DNA gyrase and topoisomerase IV. According to the docking simulation results, the three metabolites were observed to be embedded nicely within the active pocket of DNA gyrase and topoisomerase IV, as is observed by ciprofloxacin and novobiocin. The presence of the carboxylic group in helvolic acid, the sulfate group in monomethyl sulochrin-4-sulphate and the hydroxyl group in ergosterol mainly contributed to the accommodation inside the ATP-binding cavity of *S. aureus* DNA gyrase and topoisomerase IV, as well as the augmentation of the inhibitory activities that was evidenced via the development of significant H-bond interactions and thus considered novel findings as no previous study illustrated the mechanistic action of these compounds on both enzymes via a molecular docking study. Based on these results, the proposed mechanism of action for the three metabolites active against *S. aureus* enzymes, in addition to the previously studied mode of action of helvolic acid and ergosterol, allow a plausible interpretation of how these molecules carry out their antibacterial activities.

UHPLC–QTOF metabolic profiling of *A. fumigatus* ethyl acetate extract enabled us to identify antibacterial compounds as linoleic acid and oleic acid [79], fumitremorgin C [80], emodin [81], fumigallin [82], fumigaclavine C [83], hexylitaconic acid [47] and *cyclo*-(Leu-Pro) [84], which may contribute synergistically to the antibacterial activity of the compounds isolated herein. 

Collectively, this study highlights, for the first time, the detailed chemical investigation of *A. fumigatus* extract and its potential use as an antibacterial agent against *S. aureus*. In addition, we further explored the possible mechanism of actions of the secondary metabolites on DNA gyrase and topoisomerase IV via molecular docking studies. 

## 4. Materials and Methods

### 4.1. Plant Collection and Identification

Healthy leaf samples of *Albizia lucidior* (Steud.) I.C. Nielsen were collected from the Zoological garden, Giza, Egypt, in March 2019 and identified by Agr. Eng. Therese Labib, consultant of plant taxonomy at the Ministry of Agriculture and ex. Director of El-Orman Botanical Garden, Giza. A voucher specimen (no. 4.7.2019) was kept at the Herbarium of Pharmacognosy Department, Faculty of Pharmacy, and Cairo University.

The samples were stored in sterile plastic bags and kept in an icebox. The leaf samples were coded, photographed and processed for the isolation of endophytic fungi within 24 h of collection.

### 4.2. Surface Sterilization and Isolation of Endophytic Fungi

Surface sterilization of leaves was accomplished according to [63]. The efficiency of the surface sterilization procedure was ascertained for every segment of tissue [85]. Sterilized samples were cut into approximately 5 × 5 mm^2^ pieces and placed on Petri dishes that contain PDA (potato-dextrose-agar) medium supplemented with streptomycin sulfate 100 µg/mL (Sigma-Aldrich, St. Louis, MO, USA) to inhibit the growth of bacteria. The Petri dishes were sealed using Parafilm^TM^ (Parafilm^TM^, Oslo, Norway) and then incubated at 28 °C for 15 days. 

The incubated Petri dishes were checked daily for fungal colony growth. The fungal colonies originating from the inoculated plant pieces were picked and then transferred to potato dextrose agar (PDA) media, before being incubated at 28 °C to obtain a pure culture. The purified endophytic isolates were then transferred separately to PDA slants and stored at 4 °C. The isolated endophytic fungi were also maintained in a 20% glycerol stock solution and stored at −80 °C for long-term preservation.

### 4.3. Morphological and Taxonomic Identification of the Fungus

The fungal identification was carried out based on the morphological examination and the molecular taxonomy.

Morphological identification was conducted according to [86,87]. The morphological identification was based on the standard taxonomic key including texture, color and the dimensions and morphology of hyphae and conidia. The prepared slides were viewed using an image analysis system with an Olympus microscope having magnification power of 5×, 10× and 40× which is available at The Regional Center for Mycology and Biotechnology (RCMB), Al-Azhar University.

Genomic DNA was extracted using Qiagen DNeasy Mini Kit (QIAGEN GmbH, Hilden, Germany) following the manufacturer’s manual. PCR was then conducted to amplify the internal transcribed spacer (ITS) region of the extracted DNA, including the 18 s rRNA, using the universal primers ITS1 (5′-TCCGTAGGTGAACCTGCG-3′)/ ITS4 (5′-TCCTCCGCTTATTGATATGC-3′) [88]. The PCR reaction mixture (50 μL) contains 1 µg genomic DNA, 1 µL (10 μM of each primer), 1 μL of 10 mM dNTPs mixture, 5 µL of 10× standard reaction buffer, 0.25 μL of Taq DNA polymerase and nuclease-free water (up to 50 μL). PCR was performed using the following conditions: initial denaturation at 95 °C for 5 min, followed by 30 cycles of 95 °C for 30 s (denaturation), 55 °C for 30 s (annealing), 72 °C for 1 min and a final extension step of 72 °C for 5 min. The amplified products were examined by electrophoresis and sequenced in Macrogen Companies, South Korea. SeqTrace software was used to assemble sequences used for BLAST search at the NCBI (http://www.ncbi.nlm.nih.gov). Sequences alignments and phylogenetic tree were constructed using MEGA software, version 7.0 according to [89].

### 4.4. Scale-Up Fermentation and Extraction

To prepare the fungal seed culture, the endophytic strain was sub-cultured on potato dextrose agar medium plate at 28 °C for five days. Six agar plugs (0.5 × 0.5 cm^2^) with mycelia were inoculated into 250 mL Erlenmeyer flask, containing 50 mL potato dextrose broth liquid media (PDB) and incubated at 28 °C in a shaker incubator at 180 rpm for two days. An aliquot (5 mL) of fungal seed culture was transferred to 5 × 1 L Erlenmeyer flasks, each one containing 250 mL of PDB and incubated for 15 days under the same conditions of the seed culture. After incubation, the fungal broth culture (1250 mL) was extracted with ethyl acetate (1.5 L), filtered and evaporated with a rotary evaporator to yield the ethyl acetate extract (700 mg).

### 4.5. UHPLC-QTOF-MS/MS Profiling of the Fungal Crude Extract

Ultra-high-performance liquid chromatograms (UHPLC) were obtained on an Agilent LC–MS system composed of an Agilent 1290 Infinity II UHPLC coupled to an Agilent 6545 ESI-Q-TOF-MS in both negative and positive modes, aliquots (1 µL) of EtOAc extract (1 mg/mL in MeOH) were analysed on a Kinetex phenyl-hexyl (1.7 μm, 2.1 × 50 mm) column eluted with 1 min isocratic elution of 90% A (A: 100% H_2_O + 0.1% formic acid) followed by 6 min linear gradient elution to 100% B (95% MeCN + 5% H_2_O + 0.1% formic acid) with a flow rate of 0.4 mL/min. ESI conditions were set with the capillary temperature at 320 °C, source voltage at 3.5 kV and a sheath gas flow rate of 11 L/ min. Ions detected in the full scan at an intensity above 1000 counts at 6 scans/s, with an isolation width of 1.3 ~*m*/*z*, a maximum of 9 selected precursors per cycle and using ramped collision energy (5 × *m*/*z*/100 + 10 eV). Purine C_5_H_4_N_4_ [M + H]^+^ ion (*m*/*z* 121.050873) and hexakis (1H,1H,3H-tetrafluoropropoxy)-phosphazene C_18_H_18_F_24_N_3_O_6_P_3_ [M + H]^+^ ion (*m*/*z* 922.009798) were used as internal lock masses for positive mode while TFA C_2_HF_3_O_2_ [M − H]^−^ ion (*m*/*z* 112.985587) and hexakis(1H,1H,3H-tetrafluoropropoxy)-phosphazene C_18_H_18_F_24_N_3_O_6_P_3_ [M + TFA − H]^−^ ion (*m*/*z* 1033.988109) were used as internal lock masses for negative mode. The acquired MS/MS data were converted from Agilent MassHunter data files (.d) to mzXML file format using MSConvert software, part of the ProteoWizard package and transferred to the Global Natural Products Social Molecular Networking (GNPS) server (gnps.ucsd.edu).

### 4.6. Fractionation and Purification of Metabolites

The ethyl acetate extract (700 mg) was partitioned between *n*-hexane (3 × 5 mL) and 90% MeOH (10 × 5 mL). The *n*-hexane (HE) and 90% methanolic (ME) soluble extracts were evaporated to dryness, yielding 150 mg and 490 mg, respectively. 

The HE soluble extract was submitted on silica gel column chromatography (Sigma–Aldrich Chemicals, Munich, Germany) eluted with *n*-hexane: ethyl acetate mixtures at 5% increments, yielding compound ergosterol (12.5 mg, eluted at 5–10% ethyl acetate in *n*-hexane).

The ME soluble extract was fractionated by gel chromatography on a Sephadex LH-20 (Pharmacia Fine Chemicals AB, Uppsala, Sweden) with isocratic elution with MeOH. These fractions (3 mL, each) were analyzed on TLC, pre-coated silica gel 60 F 254 plates (Fluka, Sigma–Aldrich Chemicals, Munich, Germany), using methylene chloride:methanol (95:15 and 90:10, *v*/*v*) as the developing solvent. Fractions with similar chromatographic patterns were pooled together, concentrated under reduced pressure and weighed. Based on chromatographic monitoring, five fractions (1–5) were obtained. Fraction 1 (74 mg) was separated on a silica gel 60 column with a gradient elution with *n*-hexane: ethyl acetate mixtures at 5 % increments to afford two subfractions Fraction 1.1 (18 mg, eluted at 20% ethyl acetate in *n*-hexane) and Fraction 1.2 (15 mg, eluted at 35–40% ethyl acetate in *n*-hexane). Fraction 1.1 was purified by washing with methanol, leaving compound ergosterol peroxide (2.5 mg), while Fraction 1.2 was chromatographed on a silica gel 60 column with a gradient elution with methylene chloride:methanol mixtures at 0.5% increments, yielding compound helvolic acid (5.5 mg, eluted at 0.5% methanol in methylene chloride). Fraction 2 (150 mg) was purified on a silica gel 60 column with a gradient elution with *n*-hexane: ethyl acetate mixtures at 5% increments, yielding compound pseurotin A (44 mg, eluted at 45% ethyl acetate in *n*-hexane). Fraction 3 (93 mg) was subjected to chromatographic separation using preparative reversed phase HPLC (Agilent Zobrax SB-C18, 9.4 × 250 mm, 5 μm, 4 mL/min, isocratic elution 60% MeOH/H_2_O over 80 min) to yield compound monomethyl sulochrin (24 mg) at R_t_ = 60.2 min. Fraction 4 (42 mg) was chromatographed on a silica gel 60 column, using gradient elution with *n*-hexane: ethyl acetate mixtures at 5% increments to yield compound isosclerone (1.8 mg, eluted at 25% ethyl acetate in *n*-hexane) and compound monomethyl sulochrin (8 mg, eluted at 30% ethyl acetate in *n*-hexane). Fraction 5 (85 mg) was separated by RP-18 column (Sigma–Aldrich Chemicals, Munich, Germany) using water and water: methanol mixtures at 5% increments, affording compound monomethyl sulochrin-4-sulphate (3.5 mg, eluted at 25% methanol in water) and compound chaetominine (38 mg, eluted at 50% methanol in water). The structures of isolated metabolites were elucidated based on spectral data. NMR spectra were obtained on a Bruker Avance 400 spectrometer (Bruker, Yokohama, Japan) (400 MHz for ^1^H and 100 MHz for ^13^C) in the solvents indicated and referenced to residual signals (*δ*_H_ 3.31 and *δ*_C_ 49.00 ppm for MeOH, *δ*_H_ 2.50 and *δ*_C_ 39.52 ppm for DMSO, *δ*_H_ 7.26 and *δ*_C_ 77.16 ppm for CHCl_3_) in deuterated solvents. Electrospray ionization mass spectra (ESIMS) were recorded on a compact mass spectrometer (Advion, NY, USA). Optical rotation was recorded on a JASCO P-2100 polarimeter (JASCO, Tokyo, Japan) at 23.0 °C. 

### 4.7. Screening of Antimicrobial Activity

The antimicrobial activity of the fungal extract was evaluated against Gram-positive bacteria (*Bacillus subtilis* ATCC 6633 and *Staphylococcus aureus* NRRLB-767), Gram-negative bacteria isolates (*Escherichia coli* ATCC 25922, *Pseudomonas aeruginosa* ATCC 10145 and *Proteus vulgaris* ATTC 7829), one yeast (*Candida albicans* ATCC 10231) and the fungus (*Aspergillus niger* NRRLA-326). The tested organisms were obtained from the Microbiology and Immunology Department, Faculty of Medicine, Al-Azhar University, Egypt. Cultures were obtained from the culture collections of the Microbial Chemistry Department, National Research Center, Egypt.

#### 4.7.1. Agar Disc Diffusion Method

It was carried out according to the previously adopted method [90]. The diameter of inhibition zones was measured. All the experiments were carried out in triplicate, the average and standard deviation (SD) were calculated for the inhibition zone diameters. Negative controls were only treated with the respective solvents, ciprofloxacin and nystatin were used as positive controls.

#### 4.7.2. Microplate Dilution Method

This assay was performed as described by [91]. A Spectrostar Nano Microplate Reader (BMG LABTECH GmbH, Allmendgrun, Germany) was used to measure the absorbance. MIC was recorded as the lowest concentrations with no observable growth of microorganisms. Ciprofloxacin and nystatin were used as positive controls.

#### 4.7.3. In Vitro Enzyme Assessment

This assay was performed in the confirmatory diagnostic unit, Vacsera, Egypt. The screening was carried out against *S. aureus* DNA gyrase and topoisomerase IV. using *S. aureus* DNA gyrase supercoiling assay kit (Inspiralis, Norwich, UK) and *S. aureus* topoisomerase IV decatenation kit (Inspiralis) according to the protocol instructed by the manufacturer. Ciprofloxacin was used as positive control.

### 4.8. Molecular Docking Study

The molecular docking simulation was achieved using Molecular Operating Environment (MOE-Dock) software version 2014.0901 [92,93]. The X-ray crystal structures of *S. aureus* DNA gyrase and topoisomerase IV complexed with their ligands ciprofloxacin and novobiocin (PDB codes: 2XCT and 4URN) [94,95] was retrieved from Protein Data Bank. Validation of the docking procedures was performed through redocking of the original ligands and estimation of the root-mean-square deviation value (RMSD). Then, the metabolites were docked within the ATP binding sites of *S. aureus* DNA gyrase and topoisomerase IV. 

## 5. Conclusions

This study highlights the promising role of endophytic fungus *A. fumigatus*, isolated from the leaves of *A. lucidior*, as an antibacterial agent against *S. aureus*. The chemical investigation of the EtOAc extract of *A. fumigatus* has led to the isolation of eight metabolites. Additionally, the UHPLC–QTOF fingerprint represents forty-two metabolites. The fungal extract displayed potent activity against *S. aureus* via dual inhibition effects against DNA gyrase and topoisomerase IV enzymes. Furthermore, the antibacterial activities of isolated metabolites against *S. aureus* were also evaluated, yielding three metabolites—ergosterol, helvolic acid and monomethylsulochrin-4-sulphate, with considerable antibacterial activities. Therefore, these compounds were selected for the molecular docking study against *S. aureus* DNA gyrase and topoisomerase IV active sites, revealing that the active metabolites adopted the best binding style due to the presence of the tetracyclic ring system in helvolic acid and ergosterol, in addition to the carboxylic group in helvolic acid and the hydroxyl group in ergosterol, respectively, as well as the sulfate group in monomethyl sulochrin-4-sulphate. Collectively, for the first time, the study focused on the discovery of a new potent antibacterial agent, monomethyl sulochrin-4-sulphate, and its mechanistic action besides other known antibacterial metabolites.

## Figures and Tables

**Table 1:** Metabolites identified in ethyl acetate extract of *A. fumigatus* using UPLC–MS/MS.

No.	R_t_ (min)	Name	Ion *m*/*z* ppm	Molecular Formula		Fragmentation	Ref
				Positive	Negative		
1	1.92	*Cyclo-*(Leu-Pro)	211.1411	C_11_H_19_N_2_O_2_ [M + H]^+^		197.1263 183.1469	[19]
2	2.41	*Cyclo-*(Phe-Pro)	245.1266	C_14_H_17_N_2_O_2_ [M + H]^+^		217.1298 154.0699 120.0771	[20]
3	2.49	Isosclerone	357.2144	C_20_H_21_O_6_ [2M + H]^+^		339.2029 297.1932 243.1441	[21]
4	3.05	9-Deacetylfumigaclavine C	325.2252	C_21_H_29_N_2_O [M + H]^+^		307.2153 238.1444	[22]
5	3.08	Cyclotryprostatin A	410.1730		C_22_H_24_N_3_O_5_ [M − H]^−^	308.1407 293.1173	[23]
6	3.30	Fumigaclavine B	255.0293		C_16_H_19_N_2_O [M − H]^−^	237.0181 227.0344 211.0392	[24]
7	3.32	Fumigatoside F	421.1488	C_22_H_21_N_4_O_5_ [M + H]^+^		403.1451 286.0980 199.0497 120.0442	[25]
8	3.35	Pseurotin A	454.1460	C_22_H_25_NO_8_Na [M + Na]^+^		421.1453 316.0744 273.0687	[26]
9	3.52	Spirotryprostatin A	396.3100	C_22_H_26_N_3_O_4_ [M + H]^+^		340.1292 215.0815	[23]
10	3.60	Hexylitaconic Acid	213.1128		C_11_H_17_O_4_ [M − H]^−^	195.1015 169.1230 151.1132	[27]
11	3.61	Fumigaclavine C	367.2376	C_23_H_31_O_2_N_2_ [M + H]^+^		307.2177 276.1752 238.1470	[26]
12	3.64	6-Methoxyspirotryprostatin B (Spirotryprostatin G)	394.1744	C_22_H_24_N_3_O_4_ [M + H]^+^		269.1280 241.0602 213.0659	[28]
13	3.71	Tryptoquivaline F	403.1399	C_22_H_19_N_4_O_4_ [M + H]^+^		239.0816 211.0865 199.0502 171.0553 147.0551	[29]
14	3.71	Chaetominine	401.1257		C_22_H_17_N_4_O_4_ [M − H]^−^	383.1139 237.0649 145.0400	[30]
15	3.74	9-Deacetoxyfumigaclavine C	309.2312	C_21_H_29_N_2_ [M + H]^+^		278.1869 208.1018	[22]
16	3.86	Synerazol	414.1528	C_22_H_24_NO_7_ [M + H]^+^		221.0789 105.0323 77.0368	[31]
17	4.10	Azaspirofurans B	398.1227	C_21_H_20_NO_7_ [M + H]^+^		219.0641 105.0320	[32]
18	4.15	Monomethylsulochrin-4-sulphate	425.0542		C_18_H_17_O_10_S [M − H]^−^	345.0978 313.0711 181.0503	[33]
19	4.27	Fumagiringillin	475.2341		C_26_H_35_O_8_ [M − H]^−^	149.0601 131.0498 105.0708 97.0657	[34]
20	4.32	Questin	283.0607		C_16_H_11_O_5_ [M − H]^−^	283.0613 268.0373 240.0431	[26]
21	4.35	Fumitremorgin B	462.2388	C_27_H_31_N_3_O_4_ [M−H_2_O + H]^+^		394.1750 319.1750 277.1282	[26]
22	4.38	Pyripyropene A	584.2496	C_31_H_38_NO_10_ [M + H]^+^		506.2165 202.0495 148.0389	[35]
23	4.43	Azaspirofurans A	412.1392	C_22_H_22_NO_7_ [M + H]^+^		219.0587 105.0273	[32]
24	4.44	Monomethyl sulochrin	345.0978		C_18_H_17_O_7_ [M − H]^−^	313.0654 181.0446 166.0209	[29]
25	4.45	Methylorsilinate	181.0503		C_9_H_9_O_4_ [M − H]^−^	138.0318 122.0369 123.0083	[36]
26	4.55	Fumitremorgin C	380.1122	C_22_H_26_N_3_O_3_ [M + H]^+^		412.1327 380.1072 324.2825	[26]
27	4.65	Ethylα-D-glucopyranoside	209.0408	C_8_H_17_O_6_ [M + H]^+^		181.0446 165.0503	[37]
28	4.84	Emodin	269.0450		C_15_H_9_O_5_ [M − H]^−^	241.0508 225.0558 197.0603	[38]
29	4.96	6,16-*O*-Dideacetyl helvolic acid 21,16-lactone	467.2767	C_29_H_39_O_5_ [M + H]^+^		468.2348 449.2682 421.2722 135.0777	[39]
30	5.26	16-*O*-Deacetylhelvolic acid 21,16-lactone	509.2873	C_31_H_41_O_6_ [M + H]^+^		449.2567 322.2983 268.2873 135.0684	[39]
31	5.26	Helvolic acid	567.2966		C_33_H_43_O_8_ [M − H]^−^	525.2847 463.2842 403.2631 217.1227	[26]
32	5.44	16-*O*-propionyl-16-*O*-deacetylhelvolic acid/ 6-*O*-propionyl-6-*O*-deacetylhelvolic acid	581.3124		C_34_H_45_O_8_ [M − H]^−^	567.2964 441.2527 397.2271 311.1687 293.2122	[39]
33	5.45	6-*O*-propionyl-6,16-*O*-dideacetylhelvolic acid21,16-lactone	523.3043	C_32_H_43_O_6_ [M + H]^+^		449.2689 403.2631 135.0807	[39]
34	5.51	Pyripyropene F	466.2596	C_28_H_36_NO_5_ [M + H]^+^		392.2209 202.0500 148.0390	[40]
35	5.65	Pyripyropene O	508.3419		C_29_H_34_NO_7_ [M − H]^−^	464.3519 377.3212 115.0031 73.0296	[41]
36	6.09	Linoleic acid	279.2324		C_18_H_31_O_2_ [M − H]^−^	279.2325 261.2223	[42]
37	6.34	Oleic acid	281.2484		C_18_H_33_O_2_ [M − H]^−^	263.2366 240.9990	[43]
38	6.37	5,8-Epidioxyergosta-6,9(11),22-trien-3-ol	427.2474	C_28_H_43_O_3_ [M + H]^+^		409.3046 381.3149 363.2885 267.1719 147.0028	[44]
39	6.82	Ergosterol peroxide	429.3734	C_28_H_45_O_3_ [M + H]^+^		411.3615 393.3512 341.0154	[44]
40	6.90	(22E)-Ergosta4,6,8(14), 22,24(28)-pentaen-3-one	391.3007	C_28_H_39_O [M + H]^+^		267.1748 149.0235 69.0703	[45]
41	7.29	Ergosta4,6,8(14),22-tetraen-3-one	393.3165	C_28_H_41_O [M + H]^+^		268.1830 253.1595	[46]
42	7.54	Ergosterol	397.3822	C_28_H_45_O [M + H]^+^		395.3319 377.3214	[26]

**Table 2:** Antimicrobial activity of *A. fumigatus* ethyl acetate extract.

Test Microrganisms	Zone of Inhibition (ZI, mm) and Minimum Inhibitory Concentration (MIC, µg/mL)					
	Ethyl Acetate Extract		Ciprofloxacin		Nystatin	
	ZI	MIC	ZI	MIC	ZI	MIC
*S. aureus*	23.07 ± 0.51	7.81	36.90 ± 0.36	0.63	-	-
*B. subtilis*	10.63 ± 0.25	31.25	41.63 ± 0.93	0.31	-	-
*E. coli*	6.37 ± 0.32	62.50	35.40 ± 0.53	1.25	-	-
*P. aeruginosa*	NA	125.00	30.87 ± 0.35	2.50	-	-
*P. vulgaris*	14.37 ± 0.47	15.63	34.50 ± 0.40	1.25	-	-
*C. albicans*	17.13 ± 0.55	15.63	-	-	30.27 ± 0.25	2.50
*A. niger*	NA	125.00	-	-	22.27 ± 0.25	5.00

**Table 3:** Anti-*Staphylococcus aureus* activity of isolated metabolites from *A. fumigatus* ethyl acetate extract.

Compounds	Zone of Inhibition (ZI, mm)	Minimum Inhibitory Concentration (MIC, µg/mL)
Ergosterol	14.10 ± 0.30	15.63
Ergosterol Peroxide	NA	NA
Helvolic acid	33.00 ± 0.95	1.95
Pseurotin A	10.83 ± 0.21	31.25
Monomethyl sulochrin	9.90 ± 0.20	31.25
Isosclerone	NA	NA
Monomethyl sulochrin-4-sulphate	26.56 ± 0.51	3.91
Chaetominine	NA	NA
Ciprofloxacin	36.90 ± 0.36	0.63

**Table 4:** Inhibitory assay of *A. fumigatus* ethyl acetate extract against *Staphylococcus aureus* DNA gyrase and topoisomerase IV.

Sample	IC_50_ (M ± S.D.) (µg/mL)	
	DNA Gyrase	Topoisomerase IV
Ethyl acetate extract	0.86 ± 0.05	1.23 ± 0.07
Ciprofloxacin	0.51 ± 0.03	2.15 ± 0.12

IC_50_: the concentration required to produce 50% inhibition of enzyme, S.D. = standard deviation mean; each value is the mean of three values.

**Table 5:** Docking results of metabolites within the binding sites of *S. aureus* DNA gyrase.

*S. aureus* DNA Gyrase				
Compounds	Docking Score (Kcal/mol)	Amino Acid Residues (Bond Length A°)	Atoms of Compound	Type of Bond
Ciprofloxacin	−7.22	Ser1084(2.79); DG9; DG9	O(OH)(COOH); N(quinoline); Pyridine(quinoline)	H-acc Arene-cation Arene-arene
Ergosterol	−7.40	Asp1083(2.76); Asp1083(2.78); Ser1084(2.82); Ser1084(3.13)	O(OH); O(OH); O(OH); O(OH)	H-acc H-acc H-acc H-acc
Helvolic acid	−8.83	Asp508(3.38); Leu583(2.47); Gly584(2.89); His1081(2.70); Ser1085(2.72)	H(OH); O(OH); O(OH); O(CO at p-7); O(CO)(acetoxy at p-6)	H-don H-acc H-acc H-acc H-acc
Monomethyl sulochrin-4- sulphate	−7.79	Asp437(2.65); Ser438(2.48); Asp1083(2.99); Asp1083(3.46); Ser1084(3.02); Ser1084(2.54) Ser1084(2.85) DG9(2.38)	O(OH)(phenol); O(OH)(phenol); O(S=O)(OSO_3_H); H(OSO_3_H); O(S=O)(OSO_3_H); O(OH)(OSO_3_H); O(OH)(OSO_3_H); O(CO)(benzoyl)	H-acc H-acc H-don H-don H-acc H-acc H-acc H-acc

**Table 6:** Docking results of metabolites within the binding sites of *S. aureus* topoisomerase IV.

*S. aureus* Topoisomerase IV				
Compounds	Docking Score (Kcal/mol)	Amino Acid Residues (Bond Length A°)	Atoms of Compound	Type of Bond
Novobiocin	−7.60	Asn49(3.30); Asp76(2.04); Arg138(3.11); Pro82	H(OH)(oxan-4-yl); H(OCONH_2_); O(CO)(coumarin); C_6_H_2_(coumarin)	H-don H-don H-acc Arene-cation
Ergosterol	−7.92	Arg79(2.62); Arg138(3.11)	O(OH); O(OH)	H-acc H-acc
Helvolic acid	−8.43	Asn49(1.57); Asn49(2.86); Asn49(3.05); Asp52(3.01)	H(OH)(COOH); O(CO)(COOH); O(CO)(COOH); O(CO)(COOH)	H-don H-don H-don H-don
Monomethyl sulochrin-4-sulphate	−8.25	Asn49(3.59); Asp52(2.47); Arg79(2.64); Gly80(2.61); Arg138(2.93); Arg138(2.99)	H(OH)(phenol); H(OH)(phenol); O(OSO_3_H); O(S=O)(OSO_3_H); O(S=O)(OSO_3_H); O(S=O)(OSO_3_H)	H-don H-don H-acc H-don H-acc H-acc

## Data Availability

Not applicable.

## Supplementary Materials

The following supporting information can be downloaded online, phylogenetic tree and 1D NMR and selected 2D NMR spectra of isolated metabolites and tabulated 1D NMR data of monomethyl sulochrin and monomethyl sulochrin-4-sulphate. Table S1: ^1^H (400 MHz) and ^13^C (100 MHz) NMR data of compounds monomethyl sulochrin and monomethyl sulochrin-4-sulphate; Figure S1: Microscopic photo for the morphological shape of *Aspergillus* sp.; Figure S2: BLAST search (closest match) for endophytic fungus; Figure S3: Constructed phylogentic tree for endophytic fungus; Figure S4: UPLC/MS/MS chromatograms of the ethyl acetate extract of *A. fumigatus* (A; negative mode, B; positive mode); Figure S5: ESI-MS spectrum of monomethyl sulochrin-4-sulphate; Figure S6: ^13^C NMR spectrum of monomethyl sulochrin-4-sulphate (CD_3_OD); Figure S7: ^1^H NMR spectrum of monomethyl sulochrin-4-sulphate (CD_3_OD); Figure S8: HSQC spectrum of monomethyl sulochrin-4-sulphate (CD_3_OD); Figure S9: HMBC spectrum of monomethyl sulochrin-4-sulphate (CD_3_OD).
