# Supplementary material for: Identification of Antibacterial Metabolites from Endophytic Fungus Aspergillus fumigatus, Isolated from Albizia lucidior Leaves (Fabaceae), Utilizing Metabolomic and Molecular Docking Techniques
Source: Molecules. 2022 Feb 8;27(3):1117. doi: 10.3390/molecules27031117 (PMC8839868; doi:10.3390/molecules27031117)
Supplement: Supplementary file 1 [file molecules-27-01117-s001.zip › molecules-1549010 SI without track change/molecules-1549010 SI without track change.pdf]

Supporting Information

Identification of Antibacterial Metabolites from Endophytic Fungus *Aspergillus fumigatus*, Isolated from *Albizia lucidior* leaves (Fabaceae), utilizing metabolomic and molecular docking techniques

Mai E. Hussein [1,*,†], Osama G. Mohamed [1,2,†], Ahlam M. El-Fishawy [1], Hesham I. El-Askary [1], Amira S. El-Senousy [1], Ahmed A. El-Beih [3], Eman S. Nossier [4], Ahmed M. Naglah [5], Abdulrahman A. Almehizia [5], Ashootosh Tripathi [2,6] and Ahmed A. Hamed [7]

[1] Pharmacognosy Department, Faculty of Pharmacy, Cairo University, Kasr el Aini St., 11562 Cairo, Egypt; mai.husein@pharma.cu.edu.eg (M.E.H.); osama.mohamed@pharma.cu.edu.eg (O.G.M.); ahlam.elfishawy@pharma.cu.edu.eg (A.M.E.-F.); hesham.elaskary@pharma.cu.edu.eg (H.I.E.-A.); amira.elsenousy@pharma.cu.edu.eg (A.S.E.-S.)

[2] Natural Products Discovery Core, Life Sciences Institute, University of Michigan, Ann Arbor 48109, Michigan, USA; ashtri@umich.edu

[3] Department of Chemistry of Natural and Microbial Products, National Research Centre, Dokki, 12622 Giza, Egypt; aa.el-beih@nrc.sci.eg

[4] Department of Pharmaceutical Medicinal Chemistry and Drug Design, Faculty of Pharmacy (Girls), Al-Azhar University, 11754 Cairo, Egypt; dr.emannossier@gmail.com

[5] Department of Pharmaceutical Chemistry, College of Pharmacy, King Saud University, Riyadh 11451, Saudi Arabia; anaglah@ksu.edu.sa (A.M.N.); mehizia@ksu.edu.sa (A.A.A.)

[6] Department of Medicinal Chemistry, College of Pharmacy, University of Michigan, Ann Arbor 48109, Michigan, USA

[7] Microbial Chemistry Department, National Research Centre, 33 El-Buhouth Street, Dokki, 12622 Giza, Egypt; ahmedshalbio@gmail.com

* Correspondence: mai.husein@pharma.cu.edu.eg

† These authors contributed equally to this work.

Address correspondence to: Mai Emad Eldin Hussein

E-mail: mai.husein@pharma.cu.edu.eg

List of Supporting Information

Table of Contents

1. Spectroscopic characterization of Ergosterol 3
2. Spectroscopic characterization of (22E)-5α,8α-Epidioxyergosta-6,22-dien-3β-ol 3
3. Spectroscopic characterization of Helvolic acid 3
4. Spectroscopic characterization of Pseurotin A 3
5. Spectroscopic characterization of Monomethyl sulochrin 4
6. Spectroscopic characterization of Isosclerone 4
7. Spectroscopic characterization of Monomethyl sulochrin-4-sulphate 4
8. Spectroscopic characterization of Chaetominine 4

List of Tables

Table S1. ^{1}H (400 MHz) and ^{13}C (100 MHz) NMR data of compounds monomethyl sulochrin and monomethyl sulochrin-4-sulphate 5

List of Figures

Figure S1. Microscopic photo for the morphological shape of *Aspergillus* sp 6

Figure S2. BLAST search (closest match) for endophytic fungus 6

Figure S3. Constructed phylogentic tree for endophytic fungus 6

Figure S4: UPLC/MS/MS chromatograms of the ethyl acetate extract of *A. fumigatus* (A; negative mode, B; positive mode)……………………………………………………………………..7

Figure S5. ESI-MS spectrum of monomethyl sulochrin-4-sulphate.. 7

Figure S6. ^{13}C NMR spectrum of monomethyl sulochrin-4-sulphate (CD_3OD) 8

Figure S7. ^{1}H NMR spectrum of monomethyl sulochrin-4-sulphate (CD_3OD) 8

Figure S8. HSQC spectrum of monomethyl sulochrin-4-sulphate (CD_3OD)................................ 9

Figure S9. HMBC spectrum of monomethyl sulochrin-4-sulphate (CD_3OD)............................... 9

1. **Spectroscopic characterization of Ergosterol:** $C_{28}H_{44}O$,white needles, R_f : 0.89 (methylene chloride: methanol (95:5,*v/v*);^{1}H NMR ($CDCl_3$, 400 MHz): 0.63 (3H, *s*, H-18), 0.83 (6H, *t*, *J* = 6.4 Hz, H-26 and H-27), 0.92 (3H, *d*, *J* = 6.8 Hz, H-28), 0.94 (3H, *s*, H-19), 1.03 (3H, *d*, *J* = 6.6 Hz, H-21), 1.20-2.50 (m), 3.63 (1H, *tt*, *J* = 11.2, 4.2 Hz, H-3), 5.17 (1H, *dd*, *J* = 15.2, 7.8, H-23), 5.23 (1H, *dd*, *J* = 15.2, 7, H-22), 5.38 (1H, *t*, *J*= 2.7, H-6), 5.57 (1H, *dd*, *J* = 5.4, 2.1, H-7). ^{13}C NMR ($CDCl_3$, 100 MHz): 12.2 (C-18), 16.4 (C-19), 17.7 (C-28), 19.8 (C-27), 20.1 (C-26), 21.2 (C-11), 21.2 (C-21), 23.1 (C-15), 28.4 (C-16), 32.1 (C-2), 33.2 (C-25), 37.2 (C-10), 38.5 (C-1), 39.2 (C-12), 40.6 (C-20), 40.9 (C-4),43.0 (C-13), 43.0 (C-24), 46.4 (C-9), 54.7 (C-14), 55.9 (C-17), 70.6 (C-3), 116.4 (C-7), 119.7 (C-6), 132.1 (C-23), 135.7 (C-22), 139.9 (C-5), 141.5 (C-8). (EI-MS, m/z): 397 $[M+H]^+$

2. **Spectroscopic characterization of (22E)-5α,8α-Epidioxyergosta-6,22-dien-3β-ol:** R_f : 0.63 (methylene chloride: methanol (95:5,*v/v*),$C_{28}H_{44}O_3$, white powder, ^{1}H NMR ($CDCl_3$, 400 MHz): 0.81 (3H, *s*, H-18), 0.82 (3H, *d*, *J* = 6.6 Hz,, H-26), 0.84 (3H, *d*, *J* = 6.6 Hz, H-27), 0.88 (3H, *s*, H-19), 0.91 (3H, *d*, *J* = 6.8 Hz, H-28) , 1.00 (3H, *d*, *J* = 6.6 Hz, H-21), 3.97 (1H, *m*, H-3), 5.14 (1H, *dd*, *J* = 15.2, 8.1, Hz, H-23), 5.22 (1H, *dd*, *J* = 15.2, 7.4 Hz, H-22), 6.24 (1H, *d*, *J* = 8.5 Hz, H-6), 6.50 (1H, *d*, *J* = 8.5 Hz, H-7).^{13}CNMR ($CDCl_3$, 100 MHz): 13.0 (C-18), 17.7 (C-28), 18.3 (C-19), 19.8 (C-27), 20.1 (C-26), 20.8 (C-15), 21.0 (C-21), 23.6 (C-13), , 28.8 (C-16), 30.3 (C-2), 33.2 (C-25), 34.8 (C-1), 37.1 (C-10), 37.1 (C-4), 39.5 (C-12), 39.9 (C-20), 42.9 (C-24), 44.7 (C-13), 51.2 (C-9), 51.8 (C-14), 56.4 (C-17), 66.6 (C-3), 79.6 (C-8), 82.3 (C-5), 130.9 (C-7), 132.5 (C-23), 135.4 (C-22), 135.6 (C-6), (EI-MS, m/z): 429 $[M+H]^+$

3. **Spectroscopic characterization of Helvolic acid:** R_f : 0.52 (methylene chloride: methanol (95:5,*v/v*), $C_{33}H_{44}O_8$, white needles, 1H NMR ($CDCl_3$, 400 MHz): 0.93 (3H, *s*, H-18), 1.19 (3H, *s*, H-29), 1.29 (3H, *d*, *J* = 6.8 Hz, H-28), 1.45 (3H, *s*, H-19), 1.57 (1H, *m*, H-11b), 1.62 (3H, *s*, H-26), 1.70 (3H, *s*, H-27), 1.83 (1H, *d*, *J*= 3.7, H-12b), 1.91 (1H, *d*, *J*=15, H-15b), 1.96 (3H, *s*, H-31), 1.98 (1H, *m*, H-11a), 2.10 (1H, *m*, H-23b), 2.11 (3H, *s*, H-33), 2.14 (1H, *m*, H-23a), 2.27 (2H, *d*, *J* = 12.2 Hz , H-5, H-15a), 2.43 (1H,*m*, H-12a), 2.48 (2H, *m*, H-22), 2.62 (2H, *d*, *J*= 12.3, H-9, H-13), 2.78 (1H, *dq*, *J* = 12.6, 6.6 Hz, H-4), 5.11 (1H, *t*, *J* = 7.1 Hz, H-24), 5.24 (1H, *s*, H-6), 5.86 (1H, *d*, *J* = 10 Hz, H-2), 5.88 (1H, *d*, *J* = 8.1 Hz, H-16), 7.31 (1H, *d*, *J* = 10 Hz, H-1). ^{13}CNMR ($CDCl_3$, 100 MHz): 13.3 (C-28), 17.9 (C-26), 18.1 (C-18), 18.4 (C-29), 20.7 (C-31), 20.9 (C-33), 24.1(C-11), 25.9 (C-27), 26.1 (C-12), 27.7 (C-19), 28.5 (C-23), 28.8 (C-22), 38.3 (C-10), 40.6 (C-15), 40.6 (C-4), 41.8 (C-9), 46.9 (C-14), 47.4 (C-5), 49.6 (C-13), 52.8 (C-8), 73.6 (C-16), 73.9 (C-6), 122.9 (C-24), 128.0 (C-2), 130.2 (C-20), 133.1 (C-25), 147.9 (C-17), 157.3 (C-1), 169.0 (C-32), 170.3 (C-30), 174.3 (C-21), 201.5 (C-3), 208.9 (C-7).(EI-MS, m/z): 567.5 $[M–H]^–$

4. **Spectroscopic characterization of Pseurotin A:** R_f : 0.49 (methylene chloride: methanol (95:5,*v/v*), $C_{22}H_{25}NO_8$, white needles, ^{1}H NMR ($CDCl_3$, 400 MHz); 0.98 (3H, *t*, *J* = 7.5 Hz, H-15), 1.66 (3H, *s*, H-16), 2.09, 2.15 (2H, *m*, H-14a,b), 3.44 (3H, *s*, 8-OMe), 4.59 (1H, *d*, *J* = 4.4 Hz, H-10), 4.74 (1H, *dd*, *J* = 9.0, 4.4 Hz, H-11), 5.25 (1H, *dd*, *J* = 10.8, 9.0 Hz, H-12), 5.57 (1H, *dt*, *J* = 10.8, 7.5 Hz, H-13), 7.48 (2H, *t*, *J* = 7.4 Hz, H-20, H-22),7.64 (1H, *t*, *J* = 7.4 Hz, H-21), 8.32 (2H, *d*, *J* = 7.4 Hz, H-19, H-23), 8.39 (1H, *br s*, 7-NH).^{13}CNMR ($CDCl_3$, 100

MHz): 6.1 (C-16), 14.1 (C-15), 21.4 (C-14), 51.7 (8-OMe), 70.6 (C-10), 71.1 (C-11), 73.0 (C-9), 90.4 (C-8), 92.9 (C-5), 113.2 (C-3),126.5 (C-12), 128.7 (C-20, C-22), 130.8 (C-19, C-23), 132.4 (C-18), 134.8 (C-21), 136.6 (C-13), 166.7 (C-6), 185.9 (C-2), 195.4 (C-17), 196.3 (C-4). (EI-MS, m/z): 454 $[M+Na]^+$ 430 $[M-H]^-$

5. **Spectroscopic characterization of Monomethyl sulochrin :** R_f : 0.70 (methylene chloride: methanol (95:5,*v/v*), $C_{18}H_{18}O_7$, white powder, see supplementary Table 1 (EI-MS, m/z): 369 $[M+Na]^+$, 345 $[M-H]^-$

6. **Spectroscopic characterization of Isosclerone:** R_f : 0.72 (methylene chloride: methanol (95:5,*v/v*), $C_{10}H_{10}O_3$, yellowish oil, ^{1}H NMR ($CDCl_3$, 400 MHz):1.84 (1H, br *s*, 4-OH), 2.20 (1H, *m*, H-3b), 2.35 (1H, *m*, H-3a), 2.65 (1H, *ddd*, *J* = 17.8, 8.3, 4.8 Hz, H-2b), 3.01 (1H, *ddd*, *J* =17.8, 8.3, 4.8 Hz, H-2a),4.92 (1H, *dd*, *J* = 7.2, 3.5 Hz, H-4), 6.92 (1H, *d*, *J* = 7.5 Hz, H-7), 7.02 (1H, *d*, *J* = 7.5 Hz, H-5), 7.50 (1H, *t*, *J* = 7.9 Hz, H-6), 12.42 (1H, *s*, 8-OH). ^{13}CNMR ($CDCl_3$, 100 MHz): 31.4 (C-3), 34.7 (C-2), 67.9 (C-4), 115.4 (C-8a), 117.5 (C-5), 118.0 (C-7), 137.2 (C-6), 146.0 (C-4a), 162.9 (C-8), 204.3 (C-1). (EI-MS, m/z): 177 $[M- H]^-$ the optical rotation ($[\alpha]^{23}$D +11.5°)

7. **Spectroscopic characterization of Monomethyl sulochrin-4-sulphate:** R_f : 0.20 (methylene chloride: methanol (90:10,*v/v*), $C_{18}H_{18}O_{10}S$, pale red powder, see supplementary Table 1(EI-MS, m/z): 425 $[M-H]^-$

8. **Spectroscopic characterization of Chaetominine:** R_f : 0.55 (methylene chloride: methanol (95:5,*v/v*), $C_{22}H_{18}N_4O_4$, colorless crystals, ^{1}H-NMR(DMSO, 400MHz): 1.60 (3H, *d*, *J* = 6.8 Hz, H-12), 2.54 (1H, *d*, *J* = 2.6 Hz, H-13a), 2.93 (1H, *t*, *J* = 12.8 Hz, H-13b), 4.61 (1H, *q*, *J* = 6.8 Hz, H-11), 5.60 (1H, s, H-2), 5.91 (1H, *brs*, H-14), 6.70 (1H, *s*, 3-OH), 7.25 (1H, *td*, *J* = 7.6, 1.0 Hz, H-6), 7.43 (1H, *td*, *J* = 7.6, 1.0 Hz, H-7), 7.50 (2H, *t*, *J* = 7.6 Hz, H-5, H-8), 7.58 (1H, *t*, *J*=7.6 Hz, H-20), 7.69 (1H, *d*, *J*= 7.6 Hz, H-22), 7.86 (1H, *td*, *J* = 7.6, 1.0 Hz, H-21), 8.18 (1H, *d*, *J*=7.6 Hz, H-19), 8.28 (1H, *brs*, 24-NH).^{13}CNMR (DMSO, 100 MHz): 14.1 (C-12), 38.3 (C-13), 50.2 (C-14), 59.7 (C-11), 76.4 (C-3), 82.6 (C-2), 114.6 (C-8), 121.2 (C-18), 125.0 (C-5), 125.6 (C-6), 126.5 (C-19), 127.3 (C-22), 127.4 (C-20), 130.0 (C-7), 134.8 (C-21), 136.8 (C-4), 138.8 (C-9),146.8 (C-23),147.5 (C-24), 160.1 (C-15) , 165.6 (C-17), 172.1 (C-10). (EI-MS, m/z): 401 $[M-H]^-$ and 425 $[M+Na]^+$

Table S1. ^{1}H **(400 MHz) and** ^{13}C **(100 MHz) NMR data of compounds monomethyl sulochrin and monomethyl sulochrin-4-sulphate.**

**Position**	**Monomethyl sulochrin**			**Monomethyl sulochrin-4-sulphate**	
	**$CDCl_3$**	**CD_3OD**		**CD_3OD**	
	**δ_H Mult (*J* in Hz)**	**δ_H Mult (*J* in Hz)**	**δ_C**	**δ_H Mult (*J* in Hz)**	**δ_C**
**1**			128.0		132.8
**2**			158.5		157.7
**3**	6.62 (1H, *d*, *J* = 2.1 Hz)	6.68 (1H, *d*, *J* = 2.1 Hz)	104.1	7.25 (1H, *d*, *J* = 2.0 Hz)	110.1
**4**			159.8		154.6
**5**	7.02 (1H, *d*, *J* = 2.1 Hz)	6.96 (1H, *d*, *J* = 2.1 Hz)	108.8	7.51(1H, *d*, *J* = 2.0 Hz)	115.1
**6**			129.9		129.1
**7**			168.0		167.3
**8**	3.68 (3H, *s*)	3.66 (3H, *s*)	52.6	3.70 (3H, *s*)	52.7
**9**	3.71 (3H, *s*)	3.69 (3H, *s*)	56.5	3.74 (3H, *s*)	56.8
**10**			201.7		200.8
**1'**			111.8		111.5
**2'**			162.6		162.7
**3'**	6.06 (1H, br *s*)	6.21 (1H, br *s*)	104.1	6.21 (1H, br *s*)	104.1
**4'**			149.6		149.8
**5'**	6.46 (1H, br *s*)	6.39 (1H, br *s*)	111.4	6.39 (1H, br *s*)	111.4
**6'**			165.3		165.3
**7'**	3.37 (3H, *s*)	3.38 (3H, *s*)	56.3	3.36 (3H, *s*)	56.2
**8'**	2.29 (3H, *s*)	2.29 (3H, *s*)	22.4	2.30(3H, *s*)	22.4
**6'-OH**	12.97 (1H, *s*)				

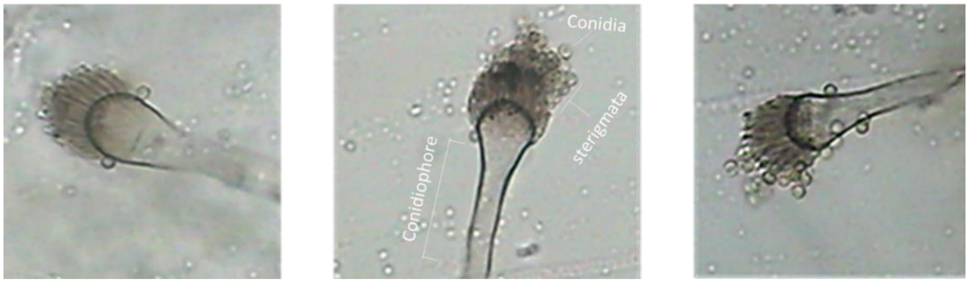

Figure S1. Microscopic photo for the morphological shape of *Aspergillus* sp.

	Description	Scientific Name	Max Score	Total Score	Query Cover	E value	Per. Ident	Acc. Len	Accession
☑	Aspergillus fumigatus strain NAUCP3 small subunit ribosomal RNA gene, partial sequence; internal transcribed s...	Aspergillus fumig...	1088	1088	99%	0.0	99.50%	602	MK334211.1
☑	Aspergillus fumigatus strain TZT-18-24 small subunit ribosomal RNA gene, partial sequence; internal transcribed ...	Aspergillus fumig...	1085	1085	99%	0.0	99.33%	601	MH919847.1
☑	Aspergillus fumigatus strain V-2 small subunit ribosomal RNA gene, partial sequence; internal transcribed spacer ...	Aspergillus fumig...	1083	1083	97%	0.0	100.00%	599	MK490926.1
☑	Aspergillus fumigatus clone SF_172 small subunit ribosomal RNA gene, partial sequence; internal transcribed sp...	Aspergillus fumig...	1081	1081	97%	0.0	100.00%	605	MT529448.1
☑	Aspergillus fumigatus strain CMXY23299 small subunit ribosomal RNA gene, partial sequence; internal transcribe...	Aspergillus fumig...	1081	1081	99%	0.0	99.33%	601	MG991645.1
☑	Aspergillus fumigatus strain S4 small subunit ribosomal RNA gene, partial sequence; internal transcribed spacer ...	Aspergillus fumig...	1081	1081	99%	0.0	99.33%	600	MW604200.1
☑	Aspergillus fumigatus isolate MEBP0074 small subunit ribosomal RNA gene, partial sequence; internal transcribe...	Aspergillus fumig...	1079	1184	100%	0.0	99.01%	728	MT597427.1
☑	Aspergillus sp. isolate AP5 small subunit ribosomal RNA gene, partial sequence; internal transcribed spacer 1, 5....	Aspergillus sp.	1079	1079	97%	0.0	99.83%	589	MN519725.1
☑	Aspergillus fumigatus isolate NIANP garden soil small subunit ribosomal RNA gene, partial sequence; internal tra...	Aspergillus fumig...	1079	1079	100%	0.0	99.01%	642	MK640673.1
☑	Aspergillus fumigatus strain SS01 18S ribosomal RNA gene, partial sequence; internal transcribed spacer 1, 5.8S...	Aspergillus fumig...	1079	1079	97%	0.0	99.83%	601	JN169122.1

Figure S2. BLAST search (closest match) for endophytic fungus

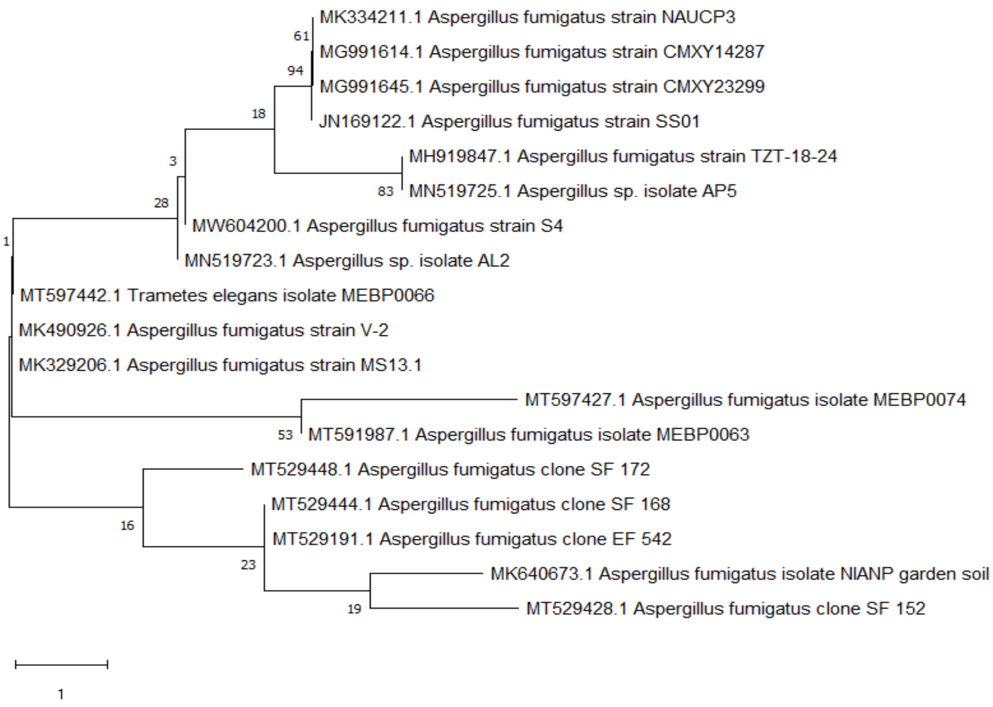

Figure S3. Constructed phylogentic tree for endophytic fungus

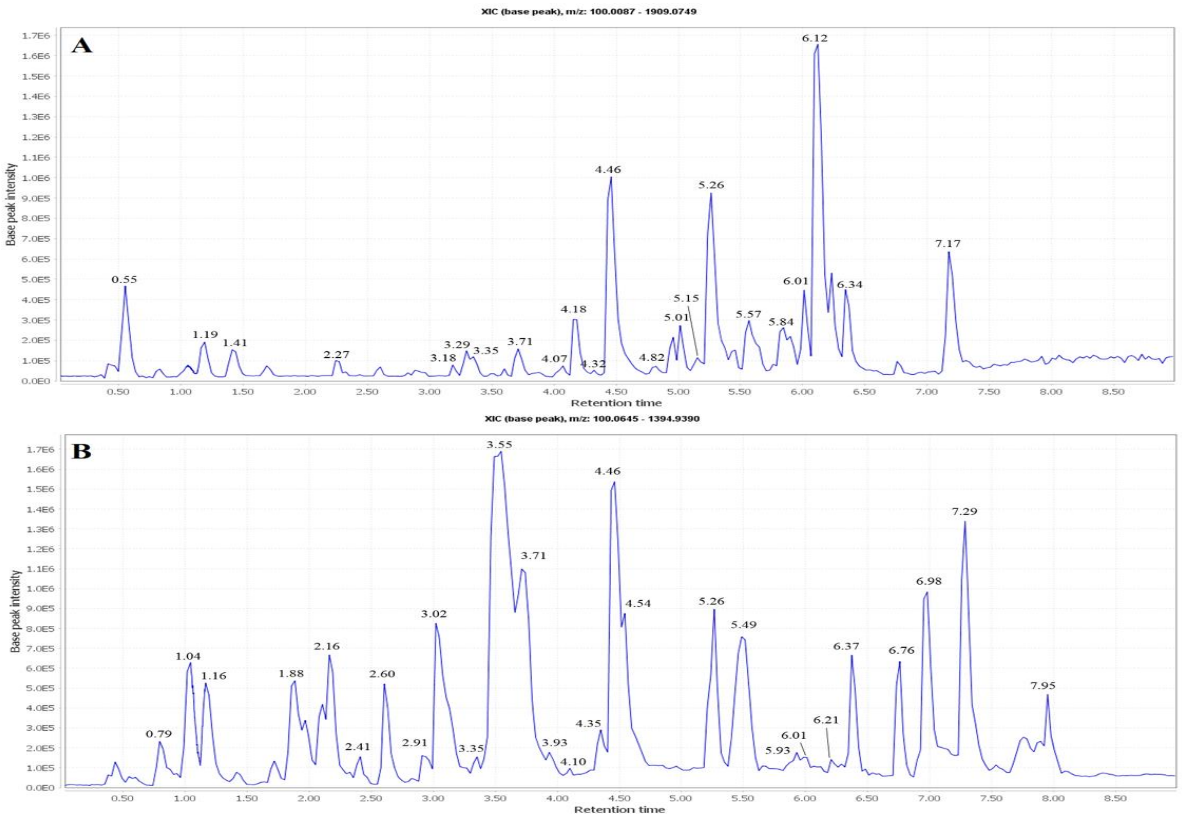

Figure S4: UPLC/MS/MS chromatograms of the ethyl acetate extract of *A. fumigatus* (A; negative mode, B; positive mode).

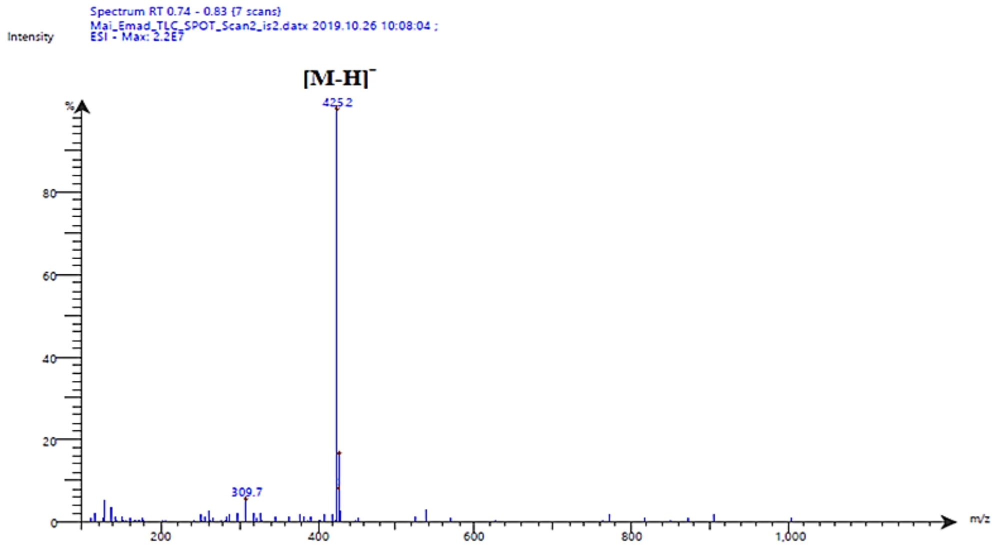

Figure S5. ESI-MS spectrum of monomethyl sulochrin-4-sulphate

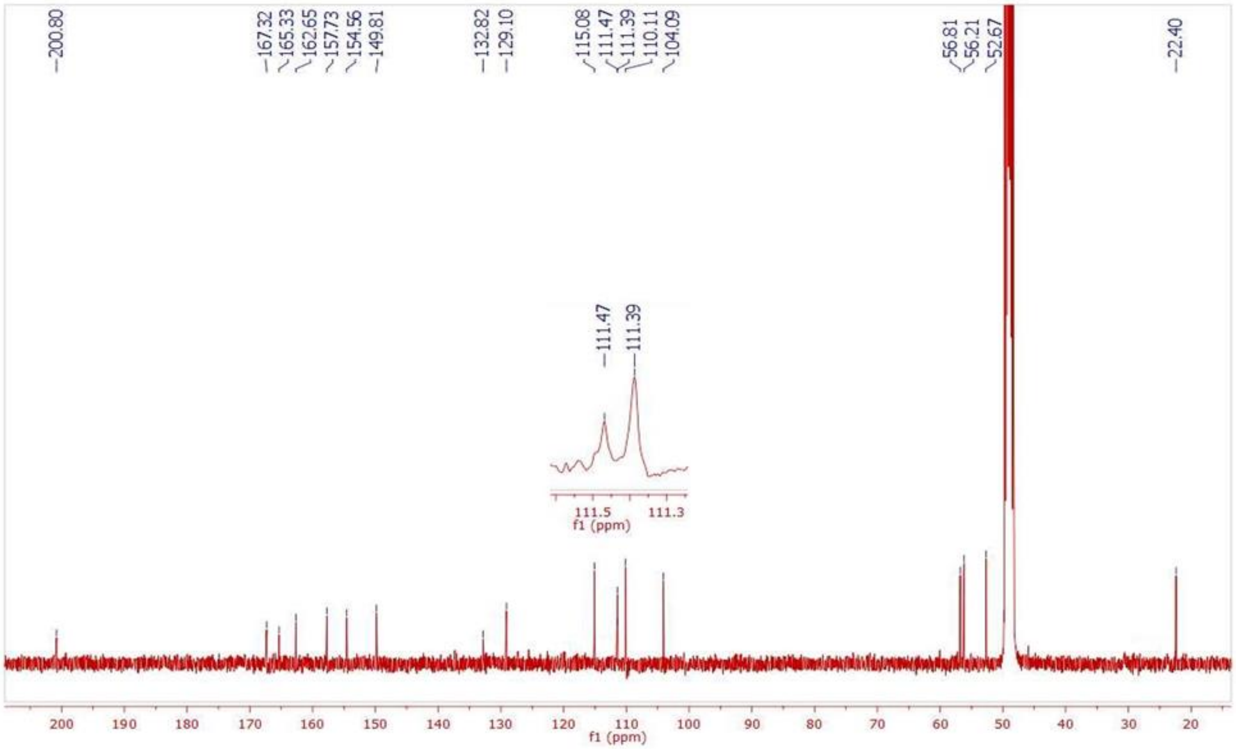

Figure S6. ^{13}C NMR spectrum of monomethyl sulochrin-4-sulphate (CD_3OD)

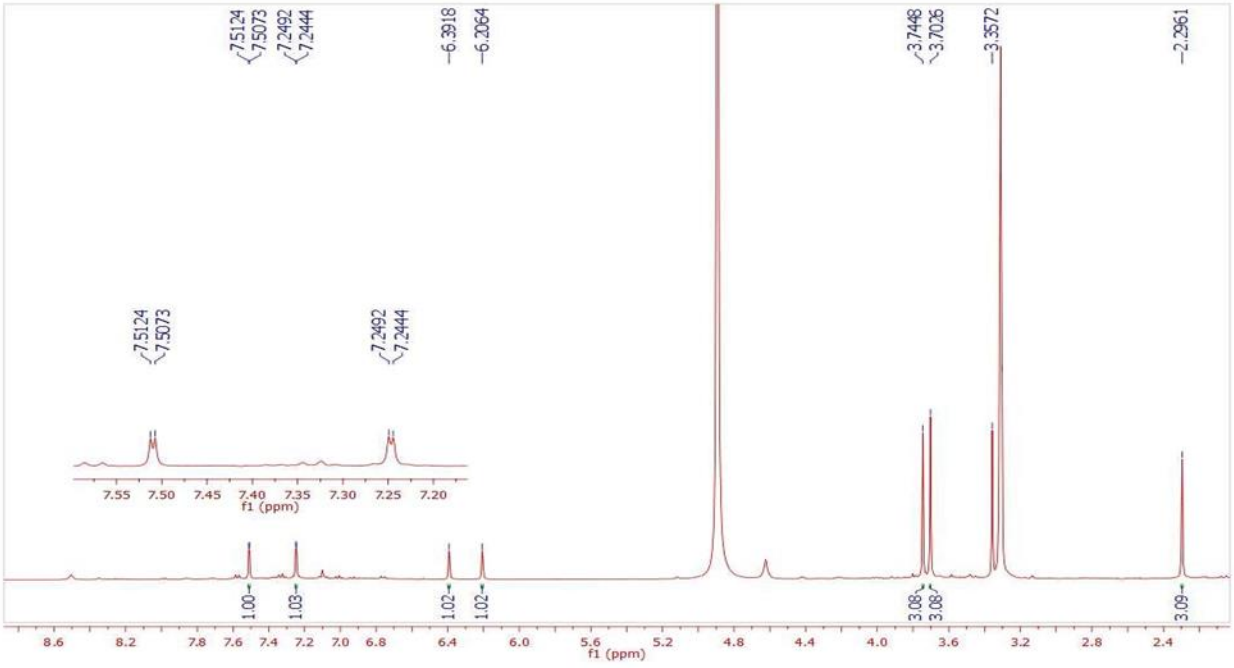

Figure S7. ^{1}H NMR spectrum of monomethyl sulochrin-4-sulphate (CD_3OD)

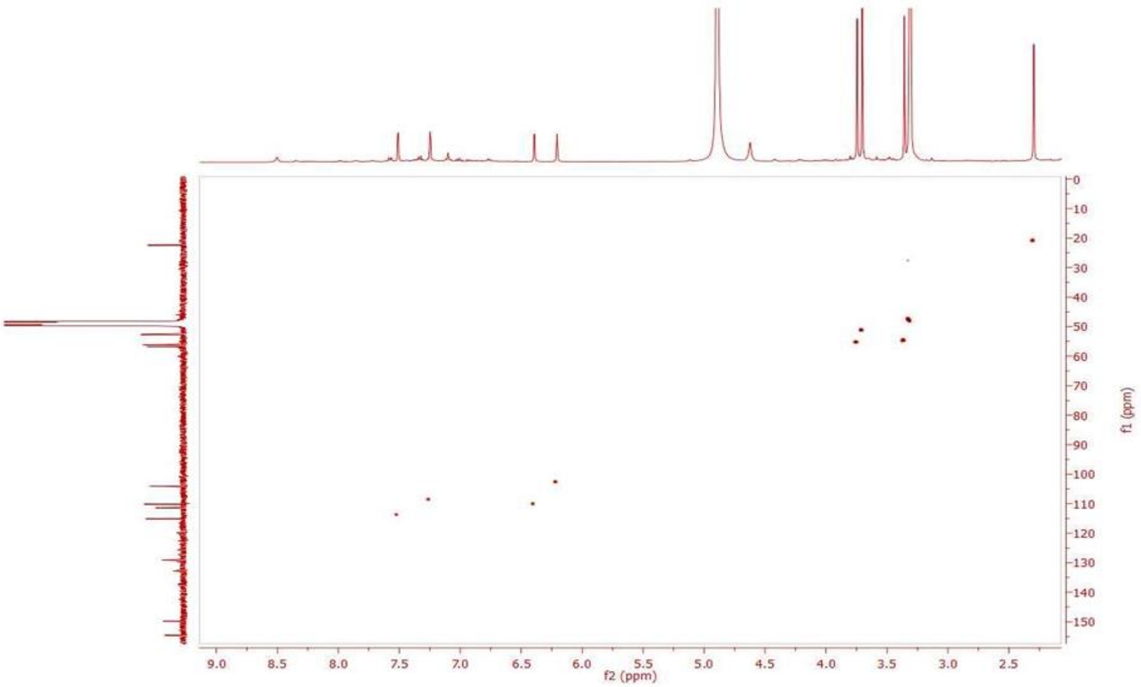

Figure S8. HSQC spectrum of monomethyl sulochrin-4-sulphate (CD_3OD)

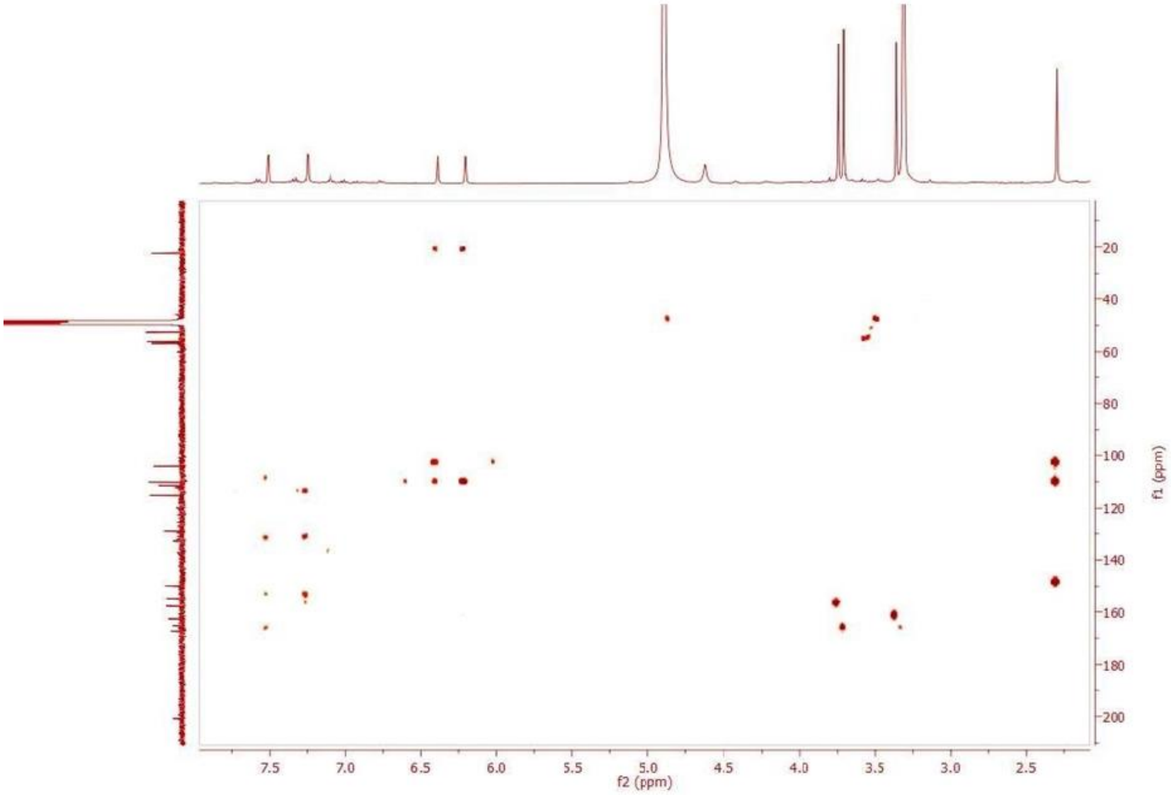

Figure S9. HMBC spectrum of monomethyl sulochrin-4-sulphate (CD_3OD)
